# A Comparative Study of Fetal and Maternal Outcomes in Registered and Unregistered Antenatal Cases in a Tertiary Care Center

**DOI:** 10.7759/cureus.66066

**Published:** 2024-08-03

**Authors:** Vidya Gaikwad, Ayushi Bhadoriya, Suhas Gaikwad

**Affiliations:** 1 Obstetrics and Gynecology, Dr. D. Y. Patil Medical College, Hospital and Research Centre, Pune, IND

**Keywords:** antenatal care, maternal and child health, maternal health, fetomaternal outcome, general obstetrics

## Abstract

Background

Antenatal care plays a crucial role in ensuring optimal maternal and neonatal health outcomes. However, disparities in access to prenatal care persist, with a subset of pregnant women failing to register for antenatal care, referred to as "unbooked" or “unregistered” pregnancies. This study aims to investigate the impact of registration status on pregnancy outcomes, considering various demographic, clinical, and socioeconomic factors. Understanding the factors influencing registration status and its implications on maternal and fetal health outcomes is essential for developing targeted interventions to improve prenatal care access and enhance overall pregnancy outcomes.

Objective

To see the difference in obstetrical complications along with feto-maternal outcomes in both registered and unregistered antenatal cases and to determine the correlation of maternal and fetal outcomes with antenatal care.

Materials and methods

This two-year observational study at Dr. D.Y. Patil Medical College's Obstetrics and Gynecology IPD in Pimpri, Pune, examined maternal and fetal outcomes in registered and unregistered pregnancies. Consent was obtained, and patients were categorized as registered and unregistered based on the number of antenatal visits. This was an observational prospective cohort study. Data on socioeconomic factors like income and education were analyzed to assess their association with registration status. Maternal outcomes included preterm delivery and complications such as preeclampsia, gestational diabetes, oligohydramnios, premature rupture of membranes, anemia, and postpartum complications. Fetal outcomes included birth weight and NICU admissions. Statistical analyses, including Chi-square tests, Fisher's exact test, and logistic regression, were used to examine relationships between variables and registration status.

Results

This study analyzed 502 cases, comprising 251 registered and 251 unregistered pregnancies, to investigate the impact of antenatal registration on maternal and fetal outcomes. Significant associations were observed between socioeconomic factors, such as lower income and education levels in unregistered pregnancies. Specifically, 46 (18.3%) unregistered cases were in the lowest income bracket, while 103 (41.0%) were in the lower-middle bracket, and the majority (132, 52.2%) had only completed secondary education. Unregistered pregnancies were linked to a higher prevalence of adverse outcomes, including preterm delivery (101, 40.23%), anemia (178, 70.9%), hypertensive disorders (30, 11.9%), gestational diabetes mellitus (16, 6.37%), fetal growth restriction (39, 15.3%), low birth weight (181, 72.1%), and NICU admissions (112, 44.6%), compared to registered pregnancies.

Conclusion

In conclusion, this study highlights the significant impact of registration status on pregnancy outcomes, emphasizing the need for comprehensive interventions to improve prenatal care access and to promote maternal and neonatal health equity. By addressing socioeconomic barriers and implementing targeted interventions, healthcare systems can strive toward ensuring optimal pregnancy outcomes for all expectant mothers. This is done by ensuring that all antenatal patients are registered for prenatal care by involving a combination of strategies focused on support, education, and accessibility.

## Introduction

Pregnancy and childbirth are universally celebrated as pivotal moments in the lives of women, families, and societies [1]. These events are integral to primary healthcare systems worldwide, highlighting the crucial importance of maternal and child health [2]. At the forefront of ensuring safe motherhood is antenatal care, which is provided by skilled health professionals to pregnant women [3]. This care encompasses health assessments, the encouragement of healthy practices, the management of complications, and the provision of social and psychological support [4].

The antenatal period emerges as a critical window to impart knowledge on birth spacing, recognized as a key determinant in improving infant survival rates [5]. A nuanced understanding of fetal growth and its intricate relationship with maternal health has prompted increased attention to the potential of antenatal care as a transformative intervention for maternal and newborn health [6]. Immunization against tetanus, prevention and treatment of malaria, management of anemia, and treatment of sexually transmitted infections (STIs) contribute significantly to improved fetal outcomes and maternal health [7]. Furthermore, the antenatal period serves as an opportune entry point for HIV prevention and care, notably in preventing mother-to-child transmission [8].

While pregnancy is a natural physiological process, it can pose risks to women's lives, occasionally culminating in maternal death [9]. Regions like Saudi Arabia still face relatively high maternal mortality rates, underscoring the critical need for effective antenatal care interventions [10]. Similarly, Sub-Saharan African countries have some of the highest maternal mortality rates in the world. Contributing factors in this region include inadequate prenatal care and a high number of births occurring outside of hospital settings [11].

The research underscores the preventable nature of a significant proportion of maternal deaths through proper management during pregnancy and labor. Adequate prenatal care emerges as a linchpin in reducing maternal and newborn mortality rates [10]. In countries with persistently high maternal and infant mortality rates, like India, grappling with issues such as maternal anemia, low birth weight, and maternal mortality becomes imperative [12,13].

Associations between maternal complications, poor perinatal outcomes, and the underutilization of antenatal and delivery care services are well-documented [14]. Additionally, socioeconomic conditions further compound these challenges, with unregistered antenatal cases often reflecting an elevated risk of adverse fetomaternal outcomes, necessitating early referral to well-equipped healthcare centers [15].

Non-utilization of antenatal care continues to be the leading cause of maternal death among women of reproductive age arising from complications of pregnancy and childbirth [16]. While the WHO recommends antenatal care, its effectiveness needs scrutiny, especially with increasing awareness among women [17]. Prenatal care booking and receipt serve as a platform for many interventions and initiatives that enhance the outcomes for both mothers and their newborns [17].

This research compares fetomaternal outcomes in registered and unregistered antenatal cases. It aims to provide a contemporary analysis of antenatal care's effectiveness in differentiating these outcomes. Understanding the impact of booking status on perinatal and maternal mortality is crucial for improving maternal and child health. By examining prenatal care practices, this study seeks to inform policies and practices for better health outcomes.

## Materials and methods

Study design and setting

This was a hospital-based observational prospective study performed at Dr. D. Y. Patil Medical College, Hospital and Research Center. This study was reviewed and approved by the Dr. D. Y. Patil University Institutional Ethics Subcommittee, with reference number: I.E.S.C./382/2022.

Participants

All pregnant women aged 19-40 years who were admitted for delivery between September 2022 and June 2024 and who consented to participate and met the inclusion criteria were included in the study. After obtaining informed consent, a demographic profile and obstetric history were taken. All cases were labeled as registered or unregistered (based on operational definition).

Inclusion and exclusion criteria

All pregnancy women who were more than 28 weeks of gestation who delivered at the institute were included. Pregnant women with multifetal pregnancy and less than 28 weeks of gestation were excluded.

Sample size

The sample size for the study was 502 patients. It was calculated by considering the proportion of eclampsia in booked and unbooked cases, which were 0.8% and 5.34%, respectively, in the study "A Comparative Study of Fetal and Maternal Outcome in Registered and Unregistered Antenatal Cases," the sample size was calculated to be 502. This calculation, which includes 251 patients in each group, was done using the software WINPEPI 11.38, assuming a significance level of 5%, a power of 80%, and a 95% confidence interval.

Study period

The study was conducted for two years from 1st September 2022 to 30th June 2024; patients present for antenatal care and admitted for delivery at Dr. D. Y. Patil Medical College, Hospital and Research Centre were studied with respect to their presentation, course, treatment, and outcomes.

Data collection and consent

The primary data source for the study comprised patients from Dr. D. Y. Patil Medical College, Hospital and Research Centre. Data collection was done after obtaining consent and using a proforma (Table 13 of Appendices) approved by the institutional ethics committee with reference number I.E.S.C./382/2022. The study primarily focused on pregnant women admitted to the labor room for delivery.

Statistical analysis

Data entry was done utilizing MS Excel (Microsoft Corporation, Redmond, Washington, United States). A statistical analysis was performed using GraphPad Prism 10. Relevant descriptive statistics were analyzed and plotted as frequency and percentage. To ascertain significant associations and patterns within the dataset, the Chi-square test and Fisher's exact test were applied wherever needed, and a p-value of 0.05 or less was considered significant.

## Results

Table 1 represents the distribution of cases based on their registration status. There were 502 cases, with an equal number of registered and unregistered cases. Specifically, 251 cases (50%) were registered, and 251 cases (50%) were unregistered, resulting in an even distribution between the two groups.

**Table 1:** Types of cases among the population

Types of cases	Cases	Percentage
Registered	251	50%
Unregistered	251	50%
Total	502	100%

Distribution of age in the population

Table 2 represents the distribution of individuals across different age groups. The age groups are categorized as 19-24, 25-34, and 35 years and above. The provided frequencies show that the largest proportion of individuals falls within the 25-34 years age range. Following closely behind is the 19-24 age group; the smallest percentage is accounted for by individuals aged 35 and above. This breakdown illustrates a demographic skew toward younger age brackets, with a substantial majority falling within the 19-34 age range.

**Table 2:** Age distribution among the population

Age group (yrs)	Frequency	Percentage
19-24	186	37.05%
25-34	286	56.97%
35 above	30	5.97%
Total	502	100.00%

Comparison of the distribution of socioeconomic status between registered and unregistered cases

The association between socioeconomic status and registration status in the study population was analyzed using the Chi-square test. It was found that the association was statistically significant (p<0.05) (Table 3). Lower socioeconomic cases were seen more in unregistered cases compared to registered cases.

**Table 3:** Distribution of socioeconomic status among the population The socioeconomic status was determined by the modified Kuppuswamy scale [18].

Socioeconomic status	Registered	Unregistered	Total	Chi-square (p-value)
Lower	11 (4.38%)	46 (18.32%)	57 (11.35%)	41.85 (<0.05)
Lower middle	97 (38.64%)	103 (41.03%)	200 (39.84%)	
Upper lower	33 (13.14%)	46 (18.32%)	79 (15.73%)	
Upper middle	96 (38.24%)	51 (20.31%)	147 (29.28%)	
Upper	14 (5.57%)	5 (1.99%)	19 (3.78%)	
Total	251	251	502	

Comparison of the distribution of educational status between registered and unregistered cases

The association between educational status and registration status in the study population was analyzed using Fisher's exact test. It was found that the association was statistically significant (p<0.05) (Table 4). Higher education levels were seen more commonly in the registered group compared to the unregistered group.

**Table 4:** Distribution of education status among the population The p-value was calculated using Fisher's exact test.

Education status	Registered	Unregistered	Total	p-value
Postgraduation	3 (1.19%)	0 (0.00%)	3 (0.59%)	<0.05
Graduation	103 (41.03%)	50 (19.92%)	153 (30.47%)	
Secondary	124 (49.40%)	132 (52.58%)	256 (50.99.%)	
Primary	20 (7.96%)	59 (23.50%)	79 (15.73%)	
Illiterate	1 (0.39%)	10 (3.98%)	11 (2.19%)	
Total	251	251	502	

Comparison of the distribution of parity status between registered and unregistered cases

Table 5 reveals a significant association between parity status and registration status, with a chi-square value of 33.84 (p<0.05). Primigravida cases, representing first-time pregnancies, were more likely to be registered, while multigravida cases showed a higher tendency to be unregistered. 

**Table 5:** Distribution of parity status among the population

Gravida	Registered	Unregistered	Total	Chi-square (p-value)
Primigravida	167 (66.53%)	102 (40.63%)	269 (53.58%)	33.84 (<0.05)
Multigravida	84 (33.46%)	149 (59.36%)	233 (46.41%)	
Total	251	251	502	

Comparison of the distribution of gestational age at delivery between registered and unregistered cases

Table 6 shows a significant association between gestational age at delivery and registration status. Preterm deliveries were more frequent among unregistered cases, while early-term deliveries were the most common and were similarly distributed between registered and unregistered cases. Fisher's exact test was applied for which the p-value <0.05, which shows a statically significant association between registration status and gestational age at delivery (Table 6).

**Table 6:** Distribution of gestational age at delivery among the population Fisher's exact test was applied for the calculation of p-value.

Gestational age at delivery	Registered	Unregistered	Total	p-value
Preterm	73 (29.08%)	101 (40.23%)	174 (34.66%)	p<0.05
Early term	100 (39.84%)	103 (41.03%)	203 (40.43%)	
Late term	77 (30.67%)	47 (18.72%)	124 (24.70%)	
Post-term	1 (0.39%)	0 (0.00%)	1 (0.19%)	
Total	251	251	502	

Comparison of the distribution of maternal antenatal complications in registered and unregistered cases

Table 7 shows that maternal antenatal complications among registered and unregistered cases reveal significant differences in certain conditions. Notably, unregistered cases exhibit a higher prevalence of complications such as fetal growth restriction (39,15.5%), premature rupture of membranes (PROM) and preterm premature rupture of membranes (PPROM) (50,19.9%), oligohydramnios (30,11.9%), and gestational diabetes mellitus(16,6.3%) compared to registered cases. However, the occurrence of gestational hypertension and its associated complications shows a similar distribution between the two groups with 25 (9.9%) registered and 30 (11.9%) unregistered cases, indicating that these conditions can occur regardless of registration status. This difference, supported by a chi-square value of 58.92 and a p-value of <0.05, shows a significant association between registration status and maternal antenatal complications. 

**Table 7:** Distribution of maternal complications among the population PROM, premature rupture of membranes; PPROM, preterm premature rupture of membranes

Maternal complications	Registered	Unregistered	Total	Chi-square (p-value)
Fetal growth restriction	10 (3.98%)	39 (15.53%)	49 (9.76%)	58.92 (<0.05)
PROM & PPROM	38 (15.13%)	50 (19.92%)	88 (17.52%)	
Oligohydramnios	9 (3.58%)	30 (11.95%)	39 (7.76%)	
Gestational diabetes mellitus	6 (2.39%)	16 (6.37%)	22 (4.38%)	
Gestational hypertension and its complications	25 (9.96%)	30 (11.95%)	55 (10.95%)	
No complication	163 (64.94%)	86 (34.26%)	249 (49.60%)	
Total	251	251	502	

Comparison of the distribution of hemoglobin levels in registered and unregistered cases

Table 8 analyzes hemoglobin (Hb) levels among 502 individuals, evenly split between registered and unregistered groups. The data reveals that individuals with normal Hb levels (>11 g/dL) are predominantly registered, while those with mild (10-10.9 g/dL) and moderate (7-9.9 g/dL) anemia are more commonly unregistered. Severe anemia (<7 g/dL) is observed only in a few unregistered cases. The Fisher's exact test shows a significant association in Hb level distribution between registered and unregistered individuals (p<0.05).

**Table 8:** Distribution of anemia among the population Fisher's exact test was applied for the calculation of the p-value.

Hb (gm/dL) level	Registered (%)	Unregistered (%)	Total (%)	p-value
>11 g/dL (normal)	165 (65.74%)	73(29.0%)	238 (47.41%)	p<0.05
10-10.9 g/dL (mild)	55 (22.23%)	85 (33.86%)	139 (27.75%)	
7-9.9 g/dL (moderate)	31 (12.35%)	87 (34.66%)	118 (23.55%)	
<7 g/dL (severe)	0 (0%)	6 (2.39%)	6 (2.39%)	
Total	251	251	502	

Comparison of the distribution of mode of delivery between registered and unregistered cases

Table 9 assesses the mode of delivery among 502 women and its distribution in registered and unregistered women. The analysis shows that vaginal deliveries were more common among registered individuals, while lower-segment cesarean sections (LSCS) were more frequent among unregistered individuals. The chi-square test indicates a significant association between registration status and the mode of delivery (p<0.05). This suggests that unregistered individuals are more likely to undergo cesarean sections, whereas registered individuals have a higher likelihood of vaginal deliveries. 

**Table 9:** Distribution of mode of delivery among the population LSCS, lower-segment cesarean section

Mode of delivery	Registered (%)	Unregistered (%)	Total (%)	Chi-square (p-value)
Vaginal delivery	108 (43.02%)	79 (31.47%)	187 (37.25%)	7.167 (p<0.05)
LSCS	143 (56.97%)	172 (68.52%)	315 (62.74%)	
Total	251	251	502	

Comparison of the distribution of postpartum complications in registered and unregistered cases

Table 10 presents the distribution of postpartum complications among registered and unregistered cases, from highest to lowest occurrence. The Fisher's exact test was done yielding a p-value <0.05, which indicates a significant association in the association of registration status across different postpartum complications. This suggests that the likelihood of experiencing postpartum complications significantly depends on whether the individual was registered for prenatal care and may rely on the health parameters and nutrition that a woman receives during her pregnancy.

**Table 10:** Distribution of postpartum complications among the population Fisher's exact test was applied for the calculation of the p-value. PPH, postpartum hemorrhage

Postpartum complications	Registered	Unregistered	Total	p-value
No complications	233 (92.82%)	207 (82.4%)	440 (87.64%)	p<0.05
Cesarean/episiotomy wound Infection	4 (1.59%)	18 (7.1%)	22 (4.38%)	
Puerperal fever	4 (1.59%)	12 (4.78%)	16 (3.18%)	
Paralytic Ileus	4 (1.59%)	6 (2.39%)	10 (1.99%)	
Postpartum hemorrhage	4 (1.59%)	6 (2.39%)	10 (1.99%)	
Peripartum cardiomyopathy	2 (0.79%)	1 (0.39%)	3 (0.59%)	
Postpartum depression	0 (0.00%)	1 (0.39%)	1 (0.19%)	
Total	251	251	502	

Comparison of the distribution of birth weights in registered and unregistered cases

Table 11 examines birth weights among 502 individuals. Birth weights below 1500 g are more common among unregistered individuals (55, 22.9%), with a similar pattern observed in the 1500-2000 g category (31, 12.3%). Conversely, birth weights above 2000 g are more frequent among registered individuals, especially in the above 2500 g category (70, 27.8%). The Chi-square test reveals a significant difference in the distribution of birth weights between registered and unregistered individuals (p<0.05). This suggests a significant association between registration status and birth weight, with registered individuals more likely to have higher birth weights.

**Table 11:** Distribution of birthweight among the population

Birth weight	Registered	Unregistered	Total	Chi-square (p-value)
<1500 g	11 (4.38%)	55 (21.91%)	66 (13.14%)	68.41 (<0.05)
1500-2000 g	22 (8.76%)	31 (12.35%)	53 (10.55%)	
2000-2500 g	64 (25.49%)	95 (37.84%)	159 (31.67%)	
Above 2500 g	154 (61.35%)	70 (27.88%)	224 (44.62%)	
Total	251	251	502	

Comparison of the distribution of NICU admissions in registered and unregistered cases

Table 12 highlights the relationship between NICU admissions and registration status. The data reveals that registered patients (61, 24.3%) have a lower rate of NICU admissions compared to unregistered patients (112, 44.6%). A significant association between NICU admissions and registration status was found with a chi-square value of 22.94 (p<0.05).

**Table 12:** Requirement of NICU among the population

NICU admission	Registered	Unregistered	Total	Chi-square (p-value)
Yes	61 (24.30%)	112 (44.62%)	173 (34.46%)	22.94 (<0.05)
No	190 (75.69%)	139 (55.37%)	329 (65.53%)	
Total	251	251	502	

## Discussion

Optimal antenatal care includes identifying high-risk pregnancies, ongoing risk assessment and ensuring a safe delivery, providing necessary follow-up care for the newborn, and supporting lactation maintenance [19]. In order to decrease peri-natal deaths and elevate the quality of care, models should have at least eight visits according to the most recent WHO standards for prenatal care, reducing the gap between registered and unregistered cases [17]. Antenatal care should commence ideally from the preconception phase when planning a pregnancy as it is a crucial period for evaluating and identifying potential risks that may negatively impact maternal and fetal health [20].

The registration status or booking status of an antenatal patient reflects the level of antenatal care she receives. Several studies have demonstrated that unbooked or unregistered mothers are at a higher risk of experiencing adverse pregnancy outcomes [21].

Our study compares outcomes between registered (booked) and unregistered (unbooked) antenatal cases, emphasizing the importance of structured and continuous prenatal care. The study offers insights into how comprehensive antenatal care can mitigate leading causes of maternal and neonatal mortality. The study included 502 cases evenly split between registered and unregistered (251 each) as shown in Table 1.

Age-wise distribution of the study population

This study (Table 2) indicates that the majority of cases, 286 (56.97%), are in the 25-34 age group. The 19-24 age group accounts for 186 (37.05%) cases, while the 35 and above age group comprises only 30 (5.98%) cases. In our study, the majority of patients who were received at the labor room for delivery were of the middle age group. Age significantly influences the status of antenatal patients, with younger women, especially teenagers, being less likely to seek prenatal care compared to older women due to reasons such as fear, lack of awareness, and socioeconomic factors. In contrast, another study showed that older women, especially those more established and educated, are more likely to understand the importance of prenatal care and have the resources and support to attend regular appointments [22].

Association of socioeconomic status and registration status

The study (Table 3) highlights the association between socioeconomic status and the registration status of delivery cases, revealing significant disparities. Lower socioeconomic groups have a higher proportion of unregistered cases, while higher socioeconomic groups show more registered cases. This suggests that individuals from lower socioeconomic backgrounds face barriers to healthcare access due to a lack of awareness or limited healthcare resources. These findings underscore the need for targeted interventions to improve healthcare registration and access among lower socioeconomic groups to ensure equitable healthcare outcomes [23]. This observation aligns with a cross-sectional study conducted in Ilorin, Nigeria, where higher household income was significantly correlated with increased antenatal care registration [24]. The study highlighted that increased income levels can motivate improved health-seeking behavior, with families more willing to invest in healthcare when financial constraints are reduced.

To increase antenatal care registration and reduce health disparities, strategies must address financial barriers faced by low-income families. Policymakers and healthcare providers should focus on initiatives like subsidies, transportation assistance, and community-based outreach programs. Reducing financial obstacles will make it easier to achieve equitable access to antenatal care, leading to improved maternal and child health outcomes.

Association of educational status and registration status

The study (Table 4) highlights significant disparities in registration status based on educational levels. Individuals with higher education, such as graduates, have a higher proportion of registered cases, while those with lower education, such as primary education or illiteracy, show more unregistered cases. These findings suggest that higher education levels are linked to better healthcare access. 

A population-based study conducted by Fagbamigbe et al. found a significant correlation between educational qualifications and the likelihood of booking for antenatal care [25]. Another study revealed that pregnant mothers with higher levels of education were statistically more likely to register for antenatal services, influenced by better health literacy and understanding of antenatal benefits [26].

Pregnant women with lower education levels face challenges in accessing care due to financial constraints or cultural barriers [27]. Public health initiatives should focus on promoting education and raising awareness about antenatal care, especially among women with lower educational backgrounds. Hence focused interventions are needed to ensure that all expectant mothers receive the necessary care as this approach reduces disparities in antenatal care, enhances maternal and neonatal health, and fosters health equity and social justice.

Association of parity status and registration status

In this study (Table 5), it was observed that the majority of cases were primigravida, accounting for 269 (53.5%) cases, with multigravida cases making up 233 (46.41%) cases. Among the registered individuals, primigravida women constituted the majority. This distribution indicates that first-time pregnant women are more likely to register for antenatal care due to increased caution, the desire to ensure their health and that of their baby, and higher awareness and education about prenatal care influenced by family, friends, and healthcare providers. The fear of unknown complications also motivates first-time mothers to seek regular medical advice.

A study conducted by Setia S. et al. also confirmed a significant correlation between parity and antenatal care registration [13]. Similarly, research by Aamir F. et al. demonstrated that primigravida women were more likely to register for antenatal care [2]. These findings highlight the importance of education and support for first-time mothers to promote antenatal care registration. It is also important for obstetricians to educate all women regardless of parity status to attend antenatal care to prevent adverse pregnancy outcomes.

Association of preterm birth and registration status

Preterm labor, defined as childbirth before 37 weeks of gestation, is significantly more prevalent among unbooked antenatal cases, raising concerns about access to prenatal care services [13]. In this study, Table 6 shows that 174 (34.6%) of total cases resulted in preterm delivery, while 327 (65.2%) delivered at term. Notably, 101(40.2%) cases of all preterm deliveries were from unregistered antenatal cases. This high rate of preterm births underscores the critical importance of effective antenatal care, as it is associated with increased neonatal morbidity and mortality.

Research by Gonied found that unbooked mothers were twice as likely to deliver preterm babies compared to their booked counterparts, highlighting a strong link between antenatal care and pregnancy duration [28]. The higher incidence of preterm births among unbooked cases is alarming due to the associated risks, such as respiratory distress syndrome, intraventricular hemorrhage, and other serious complications [29,30]. These births also place a significant burden on healthcare systems due to the need for specialized care and prolonged hospital stays [31].

Therefore, promoting full-term pregnancies is an essential role of antenatal care [32]. Regular prenatal visits enable healthcare providers to monitor fetal growth and identify risk factors that could lead to preterm labor [33]. By detecting and managing these risks early, registered mothers can receive interventions that help prevent preterm birth. 

Association of maternal antenatal complications and registration status 

The findings from our study, as presented in Table 7, underscore the critical importance of antenatal care in preventing maternal complications. Complications such as premature rupture of membranes, preterm premature rupture of membranes, gestational hypertension, and gestational diabetes were significantly more frequent among unregistered antenatal cases, with a p-value of less than 0.05 indicating a statistically significant association. Similarly, Okojie et al. (2022) found higher rates of complications like antepartum hemorrhage and gestational hypertension among unregistered patients [34]. These complications are associated with poorer outcomes for both mothers and infants, highlighting the crucial role of antenatal care in monitoring and managing pregnancy health to ensure better outcomes. Proactive antenatal care enables timely identification and intervention for potential complications, thereby improving overall pregnancy health and reducing the risks associated with maternal and infant morbidity and mortality [35].

Association of anemia and registration status

Anemia is the most prevalent hematological disorder in pregnancy, leading to increased maternal morbidity and mortality. This condition can be diagnosed and treated during the antenatal period, preventing serious complications during pregnancy and labor like preterm labor, puerperal infections, and PPH, and adverse neonatal outcomes such as low birth weight and developmental delays. Our study (Table 8) showed that anemia, which included mild and moderate anemia was seen more in unregistered cases than in registered cases, and a significant association was found between registration status and Hb levels. In another study comparing the status of early and late booking status on anemia in pregnant women, results showed that 69.7% of the women who booked late had anemia while 50.7% of those who booked early had anemia [36]. Hence providing optimum diet counseling and nutritional supplementation can prevent anemia and its complications.

Association of modes of delivery and registration status

This study shows (Table 9) that 79 (31.4%) of vaginal deliveries were unregistered and 108 (43%) were registered, totaling 187 (37.2%) cases in the population. This suggests many vaginal deliveries are unscheduled, which offers quicker recovery and lower infection risk. For LSCS, 172 (68.5%) cases were unregistered suggesting that cesarean sections are often planned for unregistered patients as they come late for antenatal visits with a lack of prenatal care, undiagnosed complications such as gestational hypertension, gestational diabetes, placental disorders like uteroplacental and fetoplacental insufficiency, anemia, and inadequate birth planning. However, there is also a substantial number of registered 143 (56.9%) cases indicating that LSCS can also occur in emergencies, possibly due to complications arising during labor like fetal distress, cord prolapse, placental abruption, and others.

Studies by AnyigorOgah CS et al. and Oraekwe OI. et al. found higher rates of cesarean sections and instrumental deliveries among women without scheduled antenatal care [37,38]. This is due to undiagnosed complications, lack of monitoring, inadequate birth planning, and unmanaged health conditions leading to emergency interventions

Association of postpartum complications among the study population

The study (Table 10) also investigated the link between booking status and postpartum complications, finding a statistically significant relationship. This result suggests due to irregular checkups, underlying health issues can go undiagnosed, and proper health education on hygiene and wound care may be missed. Additionally, socioeconomic factors in unregistered women often limit access to necessary healthcare resources, contributing to suboptimal health practices. These combined factors increase the risk of complications like wound infections, including cesarean and episiotomy wound infections, which accounted for 22 (4.3%) cases, with 18 (7.1%) unregistered and 4 (1.5%) registered cases. Other complications included puerperal fever (4 (1.59%) registered and 12 (4.7%) unregistered cases), postpartum hemorrhage (PPH) (4 (1.5%) registered and 6 (2.39%) unregistered cases), and paralytic ileus (4 (1.5%) registered and 6 (2.3%) unregistered cases). Hence to decrease postpartum complications, it is necessary to educate mothers on proper hygiene and wound care practices. Additionally, provide strong postpartum support and follow-up to promptly address any issues. In a study conducted by Chigbu et al., it was noted that unbooked mothers experienced a significantly higher incidence of pre-eclampsia/eclampsia, antepartum hemorrhage, PPH, peripartum anemia, obstructed labor, and puerperal sepsis compared to booked mothers [39].

Association of birthweight among the study population

Table 11 shows that the higher prevalence of infants with lower birth weights in unregistered cases underscores potential disparities in prenatal care access, highlighting the importance of early and consistent antenatal interventions to mitigate adverse birth outcomes [40]. In our study, a statistically significant association was found between the registration status of delivery cases and birth weight outcomes, with registered women having higher birth weights. This could be due to several factors, such as scheduled antenatal care, which allows for regular monitoring, early detection, and management of potential issues affecting fetal growth. Registered women receive essential nutritional guidance and supplements like iron and folic acid, crucial for healthy fetal development. Additionally, registration can help manage maternal conditions such as hypertension, diabetes, and anemia, which can impact fetal growth if untreated. In contrast, another study by Alamarri SS et al. showed no significant association between the booking status of pregnant women and birth weight [41]. Hence, it is necessary to further investigate the correlation between birth weight and registration status.

Association of neonatal intensive care unit admissions among the study population

The study evaluated fetal outcomes based on NICU admissions (Table 12), revealing a significant difference in NICU admission rates between registered and unregistered newborns. Unregistered newborns had a notably higher rate of NICU admissions compared to their registered counterparts. This study shows that the absence of proper antenatal care led to significantly poorer pregnancy outcomes in unregistered cases compared to registered ones. This was due to a higher incidence of maternal complications such as anemia, gestational hypertension, premature rupture of membranes, fetal growth restriction, and oligohydramnios, along with increased rates of preterm delivery and NICU admission. These findings were also confirmed by a study conducted by Aamir et al. who also found that infants born to unbooked mothers had a higher risk of NICU admission, intrauterine death, and neonatal death compared to those born to booked mothers [2]. These findings highlight the critical impact of prenatal care access on neonatal health outcomes and emphasize the need for early antenatal interventions to mitigate adverse perinatal outcomes in unbooked pregnancies and henceforth reduce NICU admission rates.

Strengths and limitations

The study's main strength is that it provides valuable insights into antenatal care's impact on maternal and neonatal health, comparing registered and unregistered cases to highlight clear differences in maternal and fetal outcomes. It uses diverse data and robust statistical tools to analyze maternal complications and neonatal outcomes, revealing significant associations like registration status and birth weight. The study also examines the influence of socioeconomic status and education on antenatal care access, emphasizing the need for enhanced services and targeted interventions for unregistered cases. These findings have important implications for healthcare policy and resource allocation, highlighting the importance of early and consistent prenatal care.

Our study was conducted at a tertiary care center, so the sampled population was only 502 cases, which might not reflect the entire population of the country. The inclusion of only patients from a specific healthcare facility may introduce selection bias, undermining the representativeness of the sample and impacting the validity of the findings. Furthermore, due to the observational nature of the study, causal relationships between variables cannot be inferred, limiting the ability to draw definitive conclusions about causation.

## Conclusions

This research highlights significant differences between registered and unregistered antenatal patients in a tertiary care setting. It shows a higher concentration of individuals aged 25-34 years and a disparity in registration status, with primigravida cases more often registered. Socioeconomic status and educational level are associated with registration status, with lower status correlating with higher rates of unregistered pregnancies. The absence of proper antenatal care leads to poorer outcomes for unregistered cases, including complications like anemia, fetal growth restriction, preterm delivery, premature rupture of membranes, oligohydramnios, gestational diabetes, gestational hypertension, and postpartum complications such as wound infection and puerperal fever. Regarding neonatal outcomes, unregistered pregnancies showed adverse trends, including higher rates of low birth weight babies and NICU admission, compared to registered pregnancies. The role of preconceptional care cannot be underestimated as it gives obstetricians the window of opportunity to detect complications such as chronic hypertension and diabetes mellitus and to optimize body mass index (BMI), correct anemia, and other health parameters.

The study underscores the critical importance of early and consistent prenatal care in improving maternal and neonatal health outcomes. Targeted interventions aimed at enhancing access to prenatal care, especially among vulnerable populations, are imperative to mitigate disparities and improve overall pregnancy outcomes.

## Appendices

**Table 13:** Proforma LSCS, lower-segment cesarean section; NICU, neonatal intensive care unit; IUGR, intrauterine growth restriction

Item	Details
Registered/unregistered	
1. Patient name	
2. Age	
3. PRN	
4. Address	
5. Obstetric score	
6. Height	
7. Weight prior to pregnancy	
8. Weight	
9. BMI	
10. Socioeconomic status	Upper/Upper middle/Lower middle/Upper lower/Lower
11. Educational status	Illiterate/primary/secondary/graduation/postgraduation
12. Presenting complaints	
13. Obstetric history	
14. Menstrual history	
15. Past history	
16. Personal history	
17. Associated co-morbidities	
18. Family history	Hypertension/diabetes mellitus/thyroid disorder/asthma/epilepsy/migraine/anemia H/o of blood transfusion or hematinic injections: Yes/No
19. General examination	Pallor, icterus, cyanosis, clubbing, lymphadenopathy, edema
20. Vital	P- BP-
21. Systemic examination	RS, CVS, CNS, and P/A
22. Obstetric examination	Fundal height, lie, presentation, liquor, FHR, uterine contraction, P/S examination, and P/V examination
23. Investigation	
24. Mode of delivery	LSCS/vaginal delivery
25. Provisional diagnosis	
26. Obstetrical risk factors	Grand multipara/pre-eclampsia/eclampsia/Rh-incompatibility/heart disease/twin pregnancy/postterm pregnancy/previous cesarean/repeated miscarriages/gestational diabetes mellitus/IUGR/premature rupture of membranes
27. Fetal outcome	Full-term birth/premature birth/intrauterine death/IUGR/fetal distress/still birth/NICU admission/neonatal death
28. Baby birth weight	
29. Postpartum period	
